# Narrowband photoblinking InP/ZnSe/ZnS quantum dots for super-resolution multifocal structured illumination microscopy enhanced by optical fluctuation

**DOI:** 10.1515/nanoph-2023-0033

**Published:** 2023-03-15

**Authors:** Liangliang Zhou, Huiqun Cao, Lilin Huang, Yingying Jing, Meiqin Wang, Danying Lin, Bin Yu, Junle Qu

**Affiliations:** Shenzhen Key Laboratory of Photonics and Biophotonics, Key Laboratory of Optoelectronic Devices and Systems of Ministry of Education and Guangdong Province, College of Physics and Optoelectronic Engineering, Shenzhen University, Shenzhen 518060, China; College of Chemistry and Environmental Engineering, Shenzhen University, Shenzhen 518060, China

**Keywords:** fluorescence, InP/ZnSe/ZnS quantum dot, optical fluctuation, photoblinking, subcellular super-resolution imaging, super-resolution multifocal structured illumination microscopy

## Abstract

Cadmium-free quantum-dot (QD) fluorophores can bridge the gap between the macroscopic and microscopic domains in fluorescence super-resolution bioimaging. InP/ZnSe/ZnS QD photoblinking fluorescent probes can improve the performance of reactive super-resolution imaging techniques and spontaneously switch fluorophores between at least two states (open and close) without depending on intense laser light and specialized buffers for bioimaging. Multifocal structured illumination microscopy (MSIM) provides a two-fold resolution enhancement in sub-diffraction imaging, but higher resolutions are limited by the pattern frequency and signal-to-noise ratio. We exploit the synergy between MSIM and spontaneously switching InP/ZnSe/ZnS QD fluorophores to further increase the imaging resolution. We demonstrate the experimental combination of optical-fluctuation-enhanced super-resolution MSIM using ultrasonic-oscillation-assisted organic solvothermal synthesis of narrowband photoblinking InP/ZnSe/ZnS QDs. The InP/ZnSe/ZnS QDs show a monodisperse grain size of approximately 9 nm, fluorescence quantum yields close to 100%, and full width at half maximum below 30 nm. The structural, electronic, and optical properties are characterized through experiments and first-principles calculations. The enhanced MSIM imaging achieves an approximate fourfold improvement in resolution for fixed cells compared with widefield imaging. The proposed InP/ZnSe/ZnS QD fluorescent probes seem promising for super-resolution imaging using MSIM.

## 1 Introduction

Over the past few decades, super-resolution fluorescence microscopy has provided a powerful tool for observing the characteristics of biological specimens [1, 2]. Several techniques, such as stimulated emission depletion microscopy and single-molecule localization microscopy using photoactivated localization and stochastic optical reconstruction [3, 4], can provide appreciable resolution but are often limited to fixed cell imaging. In addition, labeling with specific fluorophores, long acquisition times, and high-intensity laser scanning are required for imaging. Photoactivated localization requires long fluorescence emission trailing peaks for burst extinction at long wavelengths, while stochastic optical reconstruction requires fluorophore state switching (bright and dark), with a sufficiently small duty cycle often induced using intense laser light and specialized buffers or ultraviolet radiation [5, 6]. Super-resolution optical fluctuation imaging (SOFI) [7] is an alternative to single-molecule localization microscopy. It performs high-order statistical analysis of photoblinking in fluorophores over time, and the resolution increases with cumulative order *n* [8]. Hence, SOFI has higher temporal resolution, relatively insensitive labeling density, and lower phototoxicity, but higher-order resolution is limited owing to a reduction in the signal-to-noise ratio over various frames [9, 10].

A technical trade-off to handle low light while realizing high spatial and temporal resolution in 3D slicing and imaging of living cells is structured illumination microscopy (SIM) [11], which is one of the most widely used imaging methods. Spatially varying illumination modes allow to transfer high-frequency information from the sample into the detectable passband of the imaging system [12]. However, SIM imaging can only achieve twice the resolution enhancement over widefield imaging, failing to achieve the resolution requirements (20–60 nm) for nanoscopy. Currently, leveraging SIM imaging under low light can enhance the resolution to capture sample details at subwavelength scales. Complementary resolution enhancement for SOFI and SIM has been proposed without increasing the device complexity, providing insights into imaging of subwavelength cellular systems with unprecedented spatial and temporal resolution. Sroda et al. [13] proposed SOFI (second-order) enhanced image scanning microscopy to achieve resolution enhancement by an approximate factor of 2.6. Although a pinhole contributes to reducing the point-spread function, it provides a negligible improvement in resolution while increasing the acquisition time for large fields of view. Descloux et al. [12] proposed SOFI-enhanced SIM/instant SIM to increase the resolution by 2.4 times. Despite their effectiveness, Michelson SIM and flat-fielded high-throughput instant SIM require complex experimental equipment, and the resolution improvement is not considerable compared with second-order SOFI or SIM. Existing developments do not fully leverage the combination between SOFI and SIM for maximizing resolution (by around four times). Recently, multifocal SIM (MSIM) [14], a parallelized version of image scanning microscopy, has demonstrated high speed, low light power, and 3D slicing for super-resolution imaging. MSIM can achieve a twofold resolution increase and 1 Hz imaging speed. In addition, MSIM may become the mainstream super-resolution imaging method, particularly for long-term 3D super-resolution imaging of thick samples, owing to its balance between imaging depth, optical sectioning, spatiotemporal resolution, and photodamage/phototoxicity. Nevertheless, the improvement in spatial resolution achieved by MSIM is limited to a factor of 2. To further enhance the spatial resolution, we use the fluorescent molecular fluctuations in SOFI as a source of nonlinearity [15] to enhance MSIM, establishing a robust complementary mechanism.

To achieve super-resolution imaging, suitable blinking fluorescent probes for SOFI are as important as the equipment [12, 16]. Various types of photoblinking probes, including semiconductor quantum dots (QDs), organic dyes, reversible conversion fluorescent proteins, carbon nanodots, and semiconductor polymer QDs [5, 17, 18], have been used in SOFI. However, the unavailability of fluorescent probes with high quantum yield, optical stability, and biocompatibility still limits the widespread adoption of SOFI for nanoscopy. QDs such as those obtained from CdSe/CdS have been validated and used in large quantities, but they are generally toxic [19]. In addition, organic dyes often require special buffers for exhibiting fluorescent blinking properties [20]. The fluorescence lifetimes of carbon and polymer dots are approximately 0.6–7.2 ns, which is much shorter than those of QDs (∼30 ns). Short lifetimes usually interfere with cellular autofluorescence [18, 21]. To alleviate this problem, Won et al. [22] proposed high-quality InP-based QDs using an organic solvent thermal method [23, 24].

We selected the most promising fluorescent probes for biocompatible InP/ZnSe/ZnS QDs to support super-resolution fluorescence microscopy. These QDs enable high fluorescence intensity and stable anti-photobleaching properties owing to their large extinction coefficients (10–50 times higher than those of organic dyes) [25]. Theoretically, QDs with 10–20 times higher fluorescence intensity than organic dyes can achieve approximately 3–5 times higher resolution. In other words, fluorescent markers with a higher photon output can fundamentally improve the spatial resolution [26]. Tuning QD absorption, emission cross-sections, spectra (fluorescence quantum yield), fluorescence switching and blinking properties, and target specificity can facilitate high-quality super-resolution imaging. Several approaches are available to improve the QD quality, including passivation, encapsulation, and ligand exchange [22, 27]. However, toxicity and half-height width impede further development. We propose a method for synthesizing narrowband photoblinking nontoxic InP/ZnSe/ZnS QDs using an ultrasonic-oscillation-assisted organic solvent thermal method. The full width at half maximum (FWHM) (29.7 nm in 624 nm wavelength) of InP/ZnSe/ZnS QDs were reduced to less than 30 nm with a non-excellent fluorescence quantum yield. First principles revealed that the surface may induce defective (suspended bond) photoblinking switching properties, which can highlight the compatibility of SOFI with InP/ZnSe/ZnS QDs. Thus, without introducing extra workload to the experimental system, we experimentally demonstrated the improvement of the proposed fluorescent probes combined with SOFI to enhance MSIM for achieving super-resolution (∼55 nm, approximately fourfold improvement). This study revealed the importance of both sampling fluctuating scenes at multiple points in time and pixel redistribution to improve resolution. Moreover, InP/ZnSe/ZnS QDs fluorescent probes showed great potential for SOFI-enhanced MSIM (SOFI-MSIM).

## 2 Experimental section

### 2.1 Synthesis of InP/ZnSe/ZnS QDs

The synthesis of InP/ZnSe/ZnS QDs included three steps with InP, InP, and InP/ZnSe/ZnS. Figure S1a illustrates the synthesis process and depicts the core–shell-wrapped InP/ZnSe/ZnS QDs. The innermost to outermost layers were InP, ZnSe, and ZnS, and the lattice constants were 5.87, 5.67, and 5.41 Å, respectively [28]. The lattice mismatch ratio was approximately 3%, providing a theoretical basis for the synthesis of InP/ZnSe/ZnS QDs [29]. Figure S1b shows a schematic illustration of ultrasonic oscillation, which assists in obtaining more homogeneous monodisperse particles. In addition, the transient temperature effect of ultrasound enables the construction of QDs surface trap states, which are responsible for photoblinking [30]. The shell suitably coats the InP core layer with stable and controllable modulation. The characterization and analysis of InP/ZnSe/ZnS QDs are shown in Figures S2–S5.

### 2.2 InP cores

InP cores were synthesized using the one-pot method. In typical synthesis, a molar ratio of 1:3 of 0.6 mmol indium acetate (In(Ac)_3_) and 1.8 mmol palmitic acids was mixed in a 50 mL three-necked flask. The mixture was degassed and dried under vacuum (He) at 150 °C for 15 min. Then, 1.0 mmol octadecene, 0.3 mmol trimethylsilyl phosphine ((TMS)_3_P), and 0.5 mL trioctylphosphine were injected into the indium solution. After reacting at 140 °C for 1.5 min, the mixture was quickly reduced to room temperature. Finally, all solutions in the flask were drawn using a high-temperature needle.

### 2.3 InP cluster

We added 10 mL octadecene and 0.15 mmol indium acetate to another three-necked flask (100 mL). The mixture was evacuated at 220 °C for 15 min. Then, the temperature was raised to 290 °C, the InP core was quickly injected into the 100 mL three-necked flask for 15 min, and the flask was quickly cooled down to room temperature. Subsequently, slow pumping was used to inject 0.3 mmol (TMS)_3_P with 0.5 mL octadecene solution at 245 °C and 6 min/mL. After 30 min, the absorption peak of the InP cluster was approximately 575 nm, and the cluster was purified with ethyl acetate and high-purity ethanol by centrifugation.

### 2.4 InP/ZnSe/ZnS QDs

We added 10 mmol zinc acetate (Zn(Ac)_2_), 1.5 mmol lauric acid, 18 mmol oleic acid, and 20 mL octadecene to a 250 mL three-necked flask, which was degassed at 170 °C for 30 min. The color of the solution was maintained as clear as possible. Subsequently, the temperature was reduced to 110 °C. The cleaned InP cluster was added to a 250 mL three-necked flask for 5 min. Subsequently, 30 µL of HF was injected into the flask and held for 5 min, and 0.4 mmol selenium powder (Se) trioctylphosphine was quickly injected into the flask at 330 °C for 5 min. The temperature was reduced to 315 °C, and a 0.5 mmol Se-trioctylphosphine-HF solution was injected by slow pumping until reaching 5 mL and held for 10 min, in which the HF concentration was 10 µL. Then, 0.2 mL of oleic acid was quickly injected at 340 °C for 10 min. The slow pumping and oleic acid processes were performed three times. InP/ZnSe was obtained. Then, 0.5 mL 1-dodecanethiol was injected into the flask at 315 °C for 10 min. The temperature controller was then raised to 335 °C and held for 15 min. The emission wavelength stabilized at approximately 625 nm. Finally, InP/ZnSe/ZnS QDs were cleaned and vacuum dried. The InP/ZnSe/ZnS QDs (5 mg/mL) were redissolved in *n*-hexane (C_6_H_14_) and stored at 4 °C.

### 2.5 Synthesis of photoblinking InP/ZnSe/ZnS QD fluorescent probes

We sonicated 5 mg/mL InP/ZnSe/ZnS QDs at 180 W for 2 h (four times for 30 min), and the QDs were oscillated (HUXI Vortex-M) for 10 min after ultrasonication (KSJ PL-S30T, 180 W). Then, 1 mL of methanol (CH_4_O), 1 mL of chloroform (CHCl_3_), 1 mL of InP/ZnSe/ZnS QDs (5 mg/mL), 3 mL of 3-mercaptopropionic acid, and 4 mL of tetramethyl ammonium hydroxide (25% in water) were mixed in a 10 mL glass bottle. The QDs were vacuum freeze-dried after stirring for 2 h. Next, 10 µL of InP/ZnSe/ZnS QDs (5 mg/mL, 1.0 nmol), 2.4 mg of streptavidin (SA) (10 mg/mL, 42.5 nmol), 30 µL of ethyl [3-(dimethylamine) propyl] carbodiimide hydrochloride (EDC) (10 mg/mL, 1665 nmol), and 200 µL of 6.7 mM phosphate-buffered saline (PBS) (PH of 7.4) were added to a 2 mL centrifuge tube at molar ratio QDs:SA:EDC of approximately 1:40:1600. The InP/ZnSe/ZnS QDs were centrifuged in 100 KDa ultrafiltration centrifuge tubes by gently stirring for 12 h with centrifugation at 12,000 rpm for 5 min. The InP/ZnSe/ZnS QDs and SA (QD-SA fluorescent probes) were completely dissolved in 20 µL PBS (PH of 8.4) and stored at 4 °C.

### 2.6 Cellular and subcellular labeling

For microtubule labeling, African green monkey kidney cells (BS-C-1) were first cultured (37 °C, 5% CO_2_, 72 h, 20 mm glass bottom culture dishes) and washed three times with PBS (PH of 7.4) for 1 min. Second, the 1 mL 0.2% Triton X-100 permeate was added to culture dishes at 37 °C for 1 min. Then, 200 µL cytoskeleton buffer (1 × PEM, PH of 6.9 containing 0.1 M PIPES, 1 mM EGTA, and 1 mM MgCl_2_), 4% paraformaldehyde, and 0.1% glutaraldehyde were mixed in culture dishes at 37 °C for 10 min. Next, the cells were sequentially treated with 1 mL of NaBH_4_ (1 mg/mL), 200 µL of permeation solution (20 min), and 200 µL of blocking solution (40 min) and washed three times with PBS (PH of 7.4) for 5 min each time. The permeation solution (100 µL), blocking solution, and 1 µL of primary antibody (alpha-tubulin monoclonal antibody YL1/2) were mixed in culture dishes at room temperature for 1 h. Cells were washed three times with PBS and 0.5% Triton X-100 for 5 min each time. The permeation solution (100 µL), blocking solution, and 1 µL of biotinylated secondary antibody (biotin goat anti-rat antibody) were mixed in culture dishes at room temperature for 1 h. The cells were washed three times with PBS and 0.5% Triton X-100 for 5 min each time. Then, 200 µL of blocking solution and 2 µL of 40 nM QD-SA were incubated in the cells at room temperature for 1 h, and the cells were washed three times with PBS for 5 min each time. Finally, we used 1 mL of PBS (0.1% glutaraldehyde) in culture dishes for 10 min and washed three times with PBS. The cells were used with 1 mL of 50% glycerol fixed and stored at 4 °C for testing.

For mitochondrial labeling, human osteosarcoma U2OS cells were washed three times and directly fixed with 4% paraformaldehyde for 10 min. The biotinylated secondary antibody was replaced (biotin goat anti-mouse antibody). The remaining steps were the same as those used for microtubule labeling.

### 2.7 Experimental characterization equipment

The morphology, elemental composition, and size distribution of InP/ZnSe/ZnS QDs were studied by transmission electron microscopy (TEM) (JEOL-F200), atomic force microscopy (SPA-300HV). During TEM characterization, an InP/ZnSe/ZnS QD suspension was drop-cast onto an ultrathin carbon-coated holey support film with 300 mesh copper grids. The phase structure of InP/ZnSe/ZnS QDs was characterized by Raman spectroscopy (LabRAM HR Evolution) using an argon-ion laser with an excitation wavelength of 514 nm at liquid-nitrogen-controlled temperature. The optical properties of InP/ZnSe/ZnS QDs were characterized using an ultraviolet–visible spectrophotometer (PerkinElmer Lambda950) and fluorescence photoluminescence (PL) spectrometer (Horiba Fluorolog-3). Fluorescence images were collected using an objective lens (Nikon, 100 × NA = 1.35 Si oil immersion lens), sCMOS camera (Hamamatsu C5440-20UP), and *Z* piezoelectric stage (P-561.3CD). A 488 nm laser (SAPPHIRE 488-200 CW CDRH) was used to excite core–multi-shell InP/ZnSe/ZnS QDs, and a fluorescence filter (Thorlabs ET500LP) was used to detect emitted red fluorescence.

### 2.8 SOFI-MSIM data collection

To investigate the applicability of core–multi-shell InP/ZnSe/ZnS QDs as fluorescent probes for super-resolution imaging, an in-house MSIM system based on digital light innovation (DLP D4100) [31] equipped with a 100 × Si oil immersion objective (Nikon NA 1.35) was used. A 1/150× telescope was used to reduce the DMD pixel size to approximately 72 nm on the sample plane. Sparse multifocal excitation patterns were created and scanned by the DMD. In addition, a 488 nm laser was used to excite the QDs, and the fluorescence signal was captured using an sCMOS camera (Hamamatsu C5440-20UP). Optical filters were selected for fluorescence microscopy (Thorlabs ET500lp). Raw imaging data (224/120 multifocal arrays with 50/30/20 frames per array and super-resolution image) for MSIM reconstruction were acquired at 100 Hz. Core–multi-shell InP/ZnSe/ZnS QDs labeled the mitochondria and microtubules for imaging.

### 2.9 SOFI-MSIM acquisition

SOFI-MSIM comprises two main steps: Fourier ptychography SOFI [32, 33] and MSIM [14] (pixel reassignment and deconvolution). Fourier ptychography SOFI can improve the resolution by an approximate factor of 2 because it does not require the conventional SOFI high-order calculation but only the fluctuation of fluorescent molecules as scatter illumination. In addition, the MSIM resolution can be improved by more than twice through a large Stokes shift. The ratio of excitation wavelength *λ*_Ex_ to detection wavelength *λ*_Em_ is *λ*_Em_ = *k*_*e*_*λ*_Ex_ (1 < *k*_*e*_ < 2), which is *k*_*e*_ = *λ*_Em_/*λ*_Ex_ = 620/488 = 1.27. Therefore, we can obtain an improvement in resolution of more than four times.

### 2.10 Power-law distribution

The exponentially modified power-law distribution is given by [34]

(1) $$P\left(t\right)=At^{-\alpha }\exp\left(-t/\mu \right)$$

where *A* is a constant, *α* is the power exponent, and 1/*μ* is the saturation rate of the exponential distribution, which indicates the degree to which the probability density deviates from the power-law distribution.

## 3 Results and discussion

### 3.1 SOFI-MSIM system

**Figure 1:**
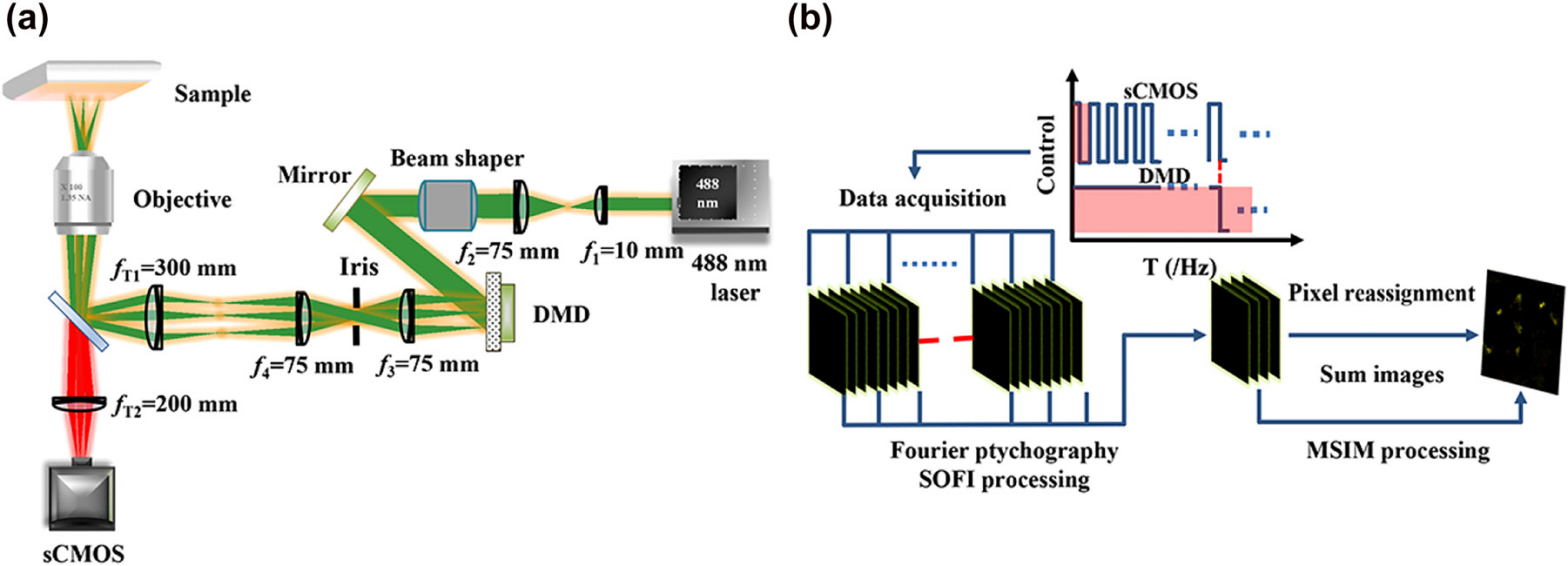
Schematics of system configuration. (a) Optical setup. (b) SOFI-MSIM process.

First, a laser beam with 488 nm wavelength was emitted through a 4*f* system consisting of lenses *f*_1_ (10 mm) and *f*_2_ (75 mm), which expanded the beam diameter by a factor of 7.5. The Gaussian beam was converted into a flat-field beam by a beam shaper, which irradiated the optical surface of a digital micromirror device (DMD) at 24° with respect to the normal of the DMD panel. The beam was reflected into a 4*f* system (*f*_3_—75 mm and *f*_4_—75 mm) by the DMD on state. An adjustable iris diaphragm was placed in the spectral plane to block the reflected light of the other diffraction orders from the DMD to avoid stray light interference. The reflected beam formed a sparse excitation array and converged on the back focal plane of the *f*_4_ lens in the 4*f* filter system through DMD modulation. Its position coincided with the front focal plane of the tube lens (*f*_T1_ of 300 mm). The optical reflective surface of the DMD was then conjugated to the front focal plane of the tube lens. Finally, the excitation array was processed using a Nikon filter box (excitation, dichromatic, and emission filters) to the sample surface and then through the tube lens (*f*_T2_ of 200 mm) to an sCMOS (scientific complementary metal–oxide semiconductor) camera (2304 × 2304 pixels, 6.5 × 6.5 µm). In the experiment, 1024 × 768 pixels were considered for the DMD, and the size of each pixel was 10.8 × 10.8 μm. The optical system was reduced to 1/150 of the original one, and the corresponding size was 72 × 72 nm on the sample surface (Figure 1a). Figure 1b illustrates the SOFI-MSIM process. SOFI-MSIM involves three postprocessing steps: data acquisition, Fourier ptychography SOFI, and MSIM. The red shadowed area of the control signal in Figure 1b shows the different exposure times of the DMD and sCMOS camera.

### 3.2 Single-particle fluorescence fluctuation of InP/ZnSe/ZnS QDs

**Figure 2:**
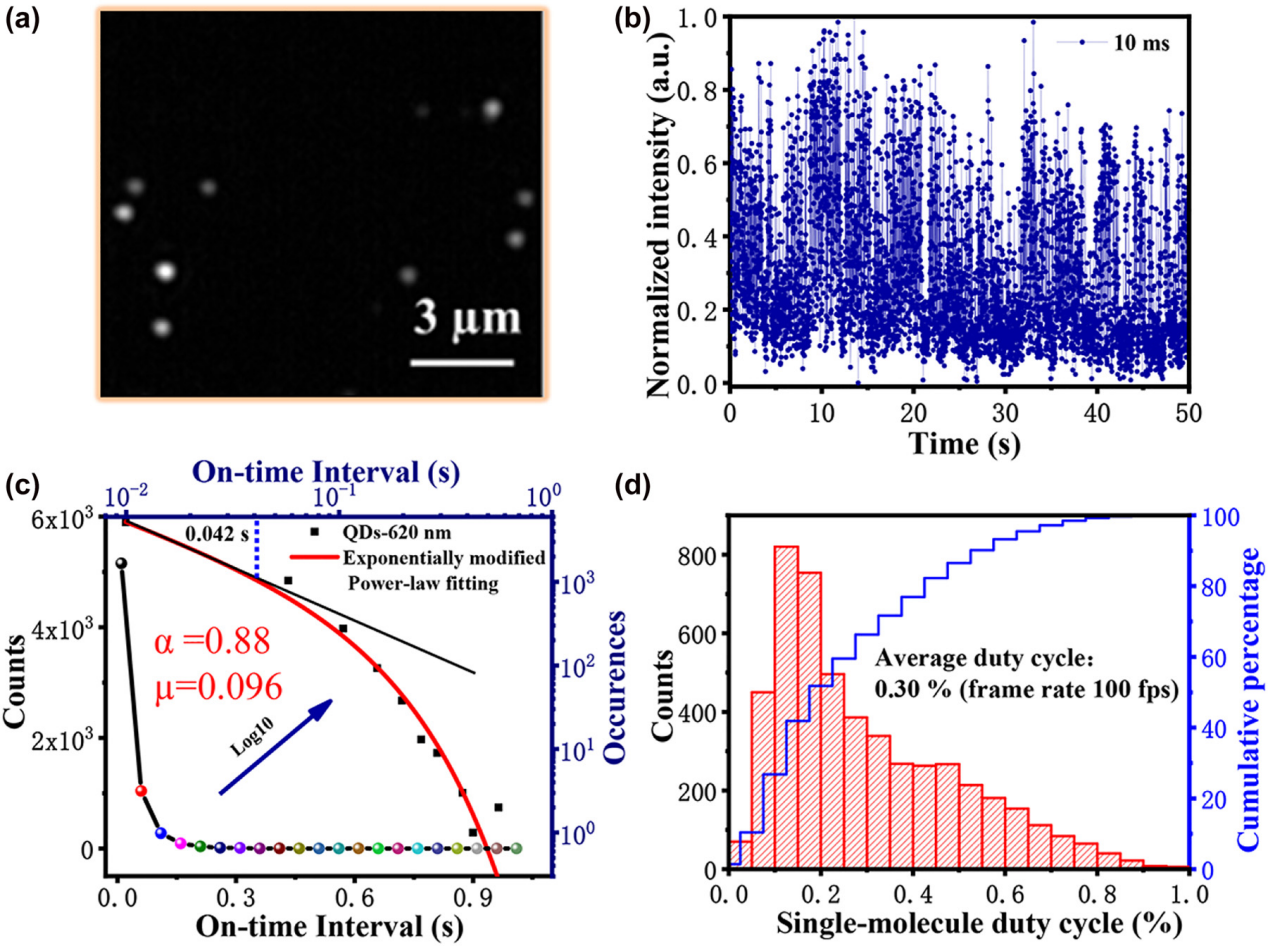
Fluorescence fluctuation of InP/ZnSe/ZnS QDs. (a) Single-particle widefield overlay of 5000 frames. (b) Photoluminescence fluctuation trace (10 ms). (c) Exponential corrected fitting of on-time interval probability density distribution of QDs. (d) Average duty cycle of QDs across several single-particle fluctuation traces.

We evaluated the fluorescence photoblinking of InP/ZnSe/ZnS QDs at the single-particle level. InP/ZnSe/ZnS QDs were excited with a 488 nm laser for MSIM widefield imaging. To highlight the single-particle imaging quality, Figure 2a shows a fluorescence image of the InP/ZnSe/ZnS QDs with no filtering. We observed bright fluorescence spots and a high signal-to-noise ratio. Figure 2b shows typical time traces of single spots for the InP/ZnSe/ZnS QD fluorescence probe for an exposure time of 10 ms. The single-particle fluorescence fluctuation for exposure times of 5 and 20 ms are shown in Figure S6a–d. The fluorescence on/off activity of the InP/ZnSe/ZnS QDs fluctuated intermittently. In addition, we calculated the fitting parameters of the probability density distributions of the on-state durations for 10 single InP/ZnSe/ZnS QDs by logarithmic transformation, obtaining the results shown in Figure 2c. Power exponent *α* = 0.88 and exponential correction *µ* = 0.096 were obtained using Equation (1) (see Experimental Section).

Hence, the bright state duration of InP/ZnSe/ZnS QDs was large, and the degree of deviation from the power-law distribution was small, being consistent with previous reports [35]. The intersection of the exponential correction and power-law curves of the probability density was defined as the average on-state duration. The blue dashed line in Figure 2c indicates that the InP/ZnSe/ZnS QDs had an average on-state duration of 42 ms. Figure 2d shows that the average duty cycle of the InP/ZnSe/ZnS QDs was 0.3% according to single-particle statistics. These results quantitatively characterized the low duty cycle of InP/ZnSe/ZnS QDs and their excellent photo-fluctuation properties, suggesting that the QDs are promising materials for super-resolution fluorescence fluctuation imaging.

### 3.3 SOFI-MSIM experiment for single InP/ZnSe/ZnS QD

**Figure 3:**
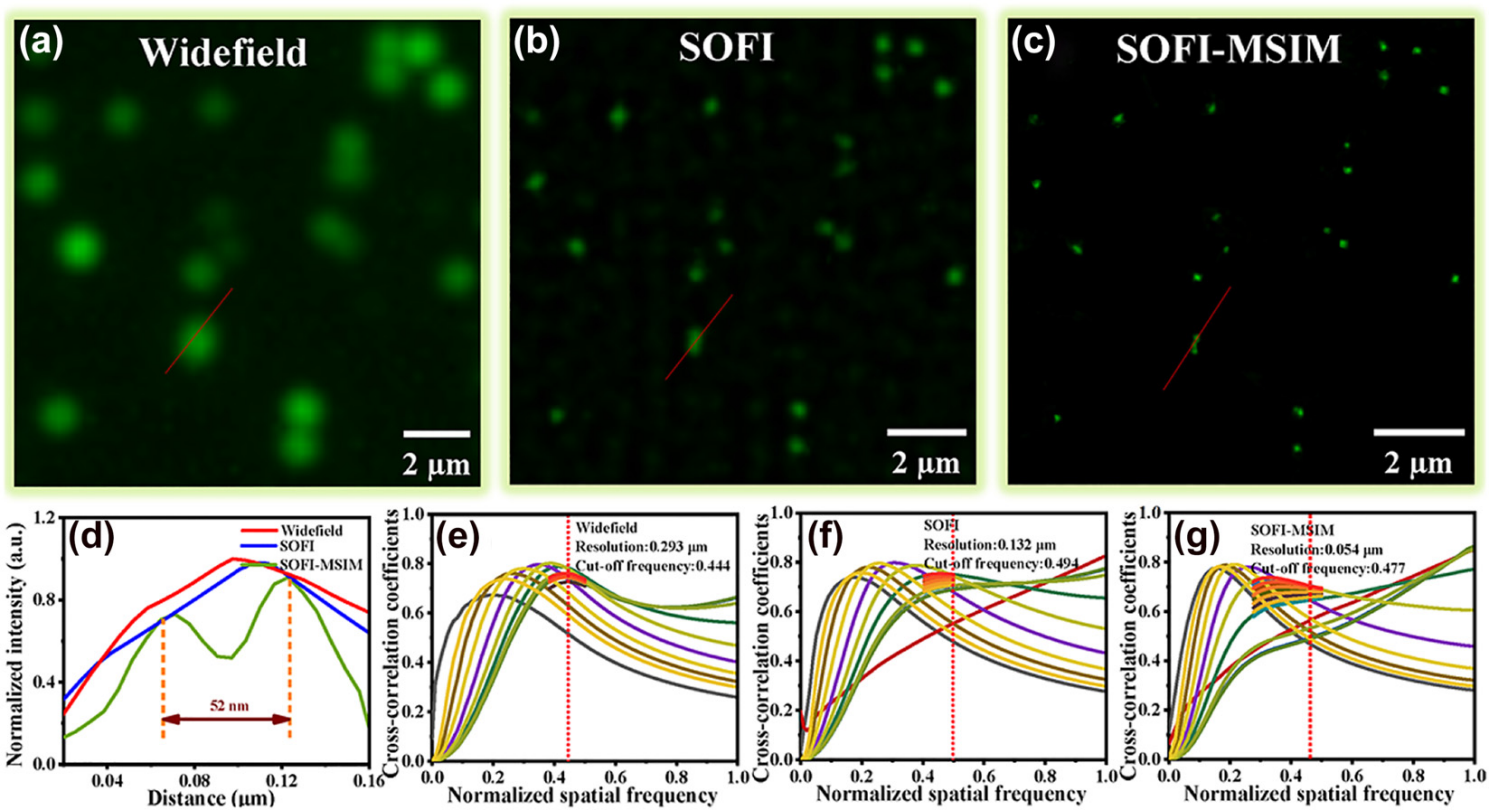
Experiment for single InP/ZnSe/ZnS QD. (a) Widefield, (b) SOFI, and (c) SOFI-MSIM images. (d) FWHM values of the two nearest points enclosed (red line). The shortest distance that could be resolved was 52 nm. (e) Resolution estimation values of widefield image (65 nm per-pixel). (f) Resolution estimation values of SOFI image (32.5 nm per-pixel). (g) Resolution estimation values of SOFI-MSIM image (13 nm per-pixel).

We analyzed and verified the effectiveness of the proposed approach. Figure 3 shows the results of the improved resolution of SOFI-MSIM imaging using a single QD. We observed resolution improvements (red line) for widefield (Figure 3a), SOFI (Figure 3b), and SOFI-MSIM (Figure 3c) images. The corresponding FWHM values (52 nm) are shown in Figure 3d. To resolution of the algorithm results was determined and analyzed. We used decorrelation analysis for parameter-free resolution estimation of a single microscopy image, namely, Resolution = 2 × pixel size/(normalized cut-off frequency) [36]. This cut-off frequency is obtained from the normalized spatial frequency. The results for widefield, SOFI and SOFI-MSIM images were 293, 132 and 54 nm, respectively (Figure 3e–g). According to the Abbe limit, the widefield resolution was approximately 231 nm, being improved by an approximate factor of 4.2 when using SOFI-MSIM, indicating optimal compliance consistent with the theoretical analysis (see Experimental Section).

### 3.4 SOFI-MSIM imaging of biological sample

**Figure 4:**
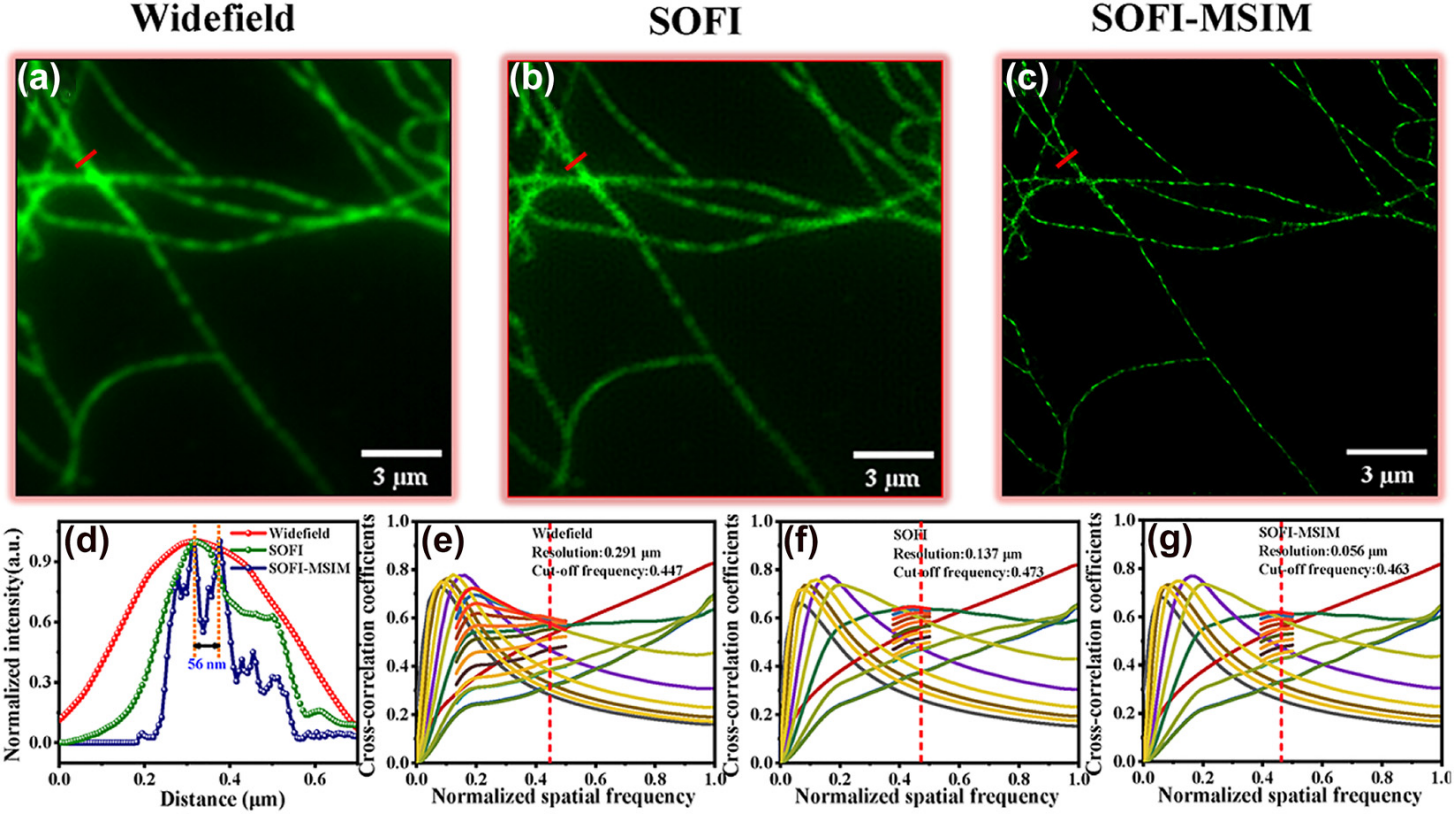
Microtubule super-resolution imaging. (a) Widefield, (b) SOFI, and (c) SOFI-MSIM images. (d) Comparison of blue line segments in microtubule after processing using widefield imaging, SOFI, and SOFI-MSIM to distinguish spectrum (52 nm). (e) Resolution estimation values of widefield image (65 nm per-pixel). (f) Resolution estimation values of SOFI image (32.5 nm per-pixel). (g) Resolution estimation values of SOFI-MSIM image (13 nm per-pixel).

We demonstrated SOFI-enhanced MSIM in fixed BS-C-1 cell samples using a 488 nm laser for microtubule imaging. Figure 4a–c show the image processing of microtubules for widefield imaging, SOFI, and SOFI-MSIM, respectively. We analyzed the effect of resolution (∼56 nm) on the normalized spectrum, as shown in Figure 4d. The fine structure of each microtubule was observed more intuitively using SOFI-MSIM. We also performed decorrelation analysis for parameter-free resolution estimation of a single microscopy image, obtaining the results shown in Figure 4e–g, respectively. The resolution estimation values were 291 nm for widefield, 137 nm for SOFI, and 56 nm for SOFI-MSIM, indicating an approximate fourfold resolution increase. Hence, the resolution was substantially increased without requiring buffers and with very high power densities.

**Figure 5:**
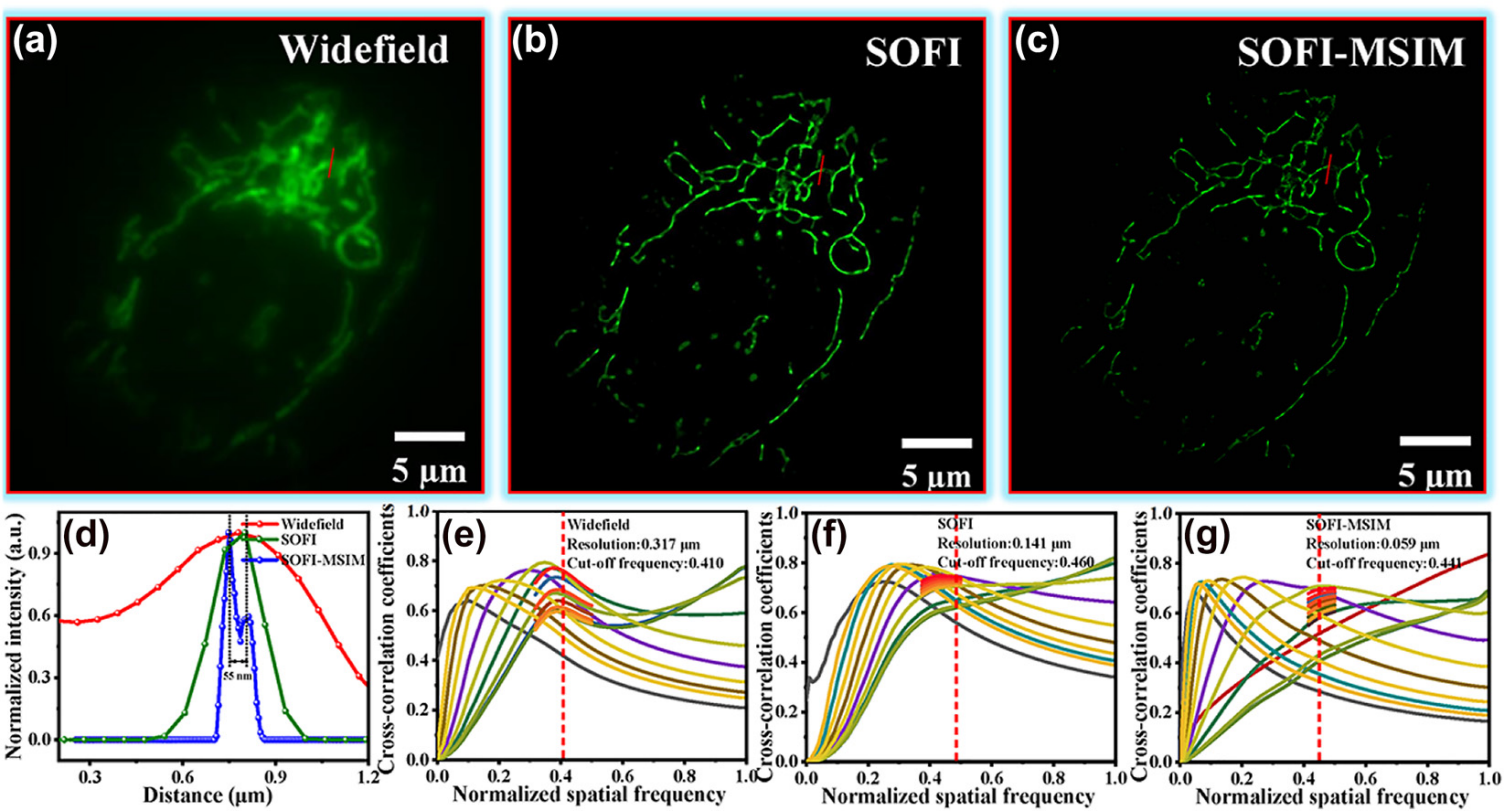
Mitochondria super-resolution imaging. (a) Widefield, (b) SOFI, and (c) SOFI-MSIM images. (d) Comparison of blue line segments in mitochondria after processing using widefield imaging, SOFI, and SOFI-MSIM to distinguish spectrum (55 nm). (e) Resolution estimation values of widefield image (65 nm per-pixel). (f) Resolution estimation values of SOFI image (32.5 nm per-pixel). (g) Resolution estimation values of SOFI-MSIM image (13 nm per-pixel).

In addition, we demonstrated the enhancement in mitochondrial resolution for fixed U2OS cells (Figure 5a–c). We obtained mitochondrial images processed by widefield imaging, SOFI, and SOFI-MSIM. The resolution of mitochondria could be improved to approximately 55 nm (red line), as shown in Figure 5d. We also performed decorrelation analysis for parameter-free resolution estimation of a single microscopy image. Figure 5e–g respectively show that the mitochondria provided resolution estimation values of 317 nm for the widefield image, 141 nm for the SOFI image, and 59 nm for the SOFI-MSIM image. The results indicate an approximate resolution increase by four times when using SOFI-MSIM. In addition, multifocal arrays can effectively improve the signal-to-noise ratio in a widefield, thus reducing the inter-array spacing. Therefore, the number of acquired frames can also improve the resolution by approximately four times. To evaluate this concept, we used 120 multifocal arrays with different frame accumulations (50, 30, and 20) per array for SOFI-MSIM reconstruction. The imaging improvements were verified for both microtubules and mitochondria, as shown in Figures S7 and S8, respectively. Hence, the SOFI-MSIM combination improved image resolution for microtubule or mitochondria imaging compared with SOFI or MSIM alone (see Figure S9).

## 4 Conclusions

In summary, we demonstrated efficient modulation of photoblinking of narrowband (29.7 nm) InP/ZnSe/ZnS QDs and achieved super-resolution subcellular imaging. Based on our characterization results, we developed a strategy for controlled synthetic photoblinking of InP/ZnSe/ZnS QDs probes by ultrasonic-oscillation-assisted organic solvothermal method, which may influence fluorescence blinking through the simulated energy band gap and optical absorption spectrum by first principles. The resulting photoblinking behavior shows great potential for realizing SOFI-enhanced MSIM nanoscopy. In typical InP/ZnSe/ZnS QDs -labeled microtubule and mitochondria structures, we achieve an approximate fourfold (∼55 nm) super-resolution improvement in SOFI-MSIM compared with widefield imaging. We consider the presented implementations a promising alternative for enhancing the resolution in biological imaging to investigate subcellular dynamics and subtle structural information beyond the diffraction limit.

## Supplementary Material

Supplementary Material Details

Supplementary Material Details
