# Supplementary material for: Narrowband photoblinking InP/ZnSe/ZnS quantum dots for super-resolution multifocal structured illumination microscopy enhanced by optical fluctuation
Source: Nanophotonics. 2023 Mar 15;12(9):1777–85. doi: 10.1515/nanoph-2023-0033 (PMC11501639; doi:10.1515/nanoph-2023-0033)
Supplement: Supplementary file 2 — Supplementary Material Details [file j_nanoph-2023-0033_suppl_002.pdf]

Supporting Information

Liangliang Zhou, Huiqun Cao*, Lilin Huang, Yingying Jing, Meiqin Wang, Danying Lin*, Bin Yu* and Junle Qu

Narrowband Photoblinking InP/ZnSe/ZnS Quantum Dots for Super-Resolution Multifocal Structured Illumination Microscopy Enhanced by Optical Fluctuation

The synthesis of InP/ZnSe/ZnS QDs

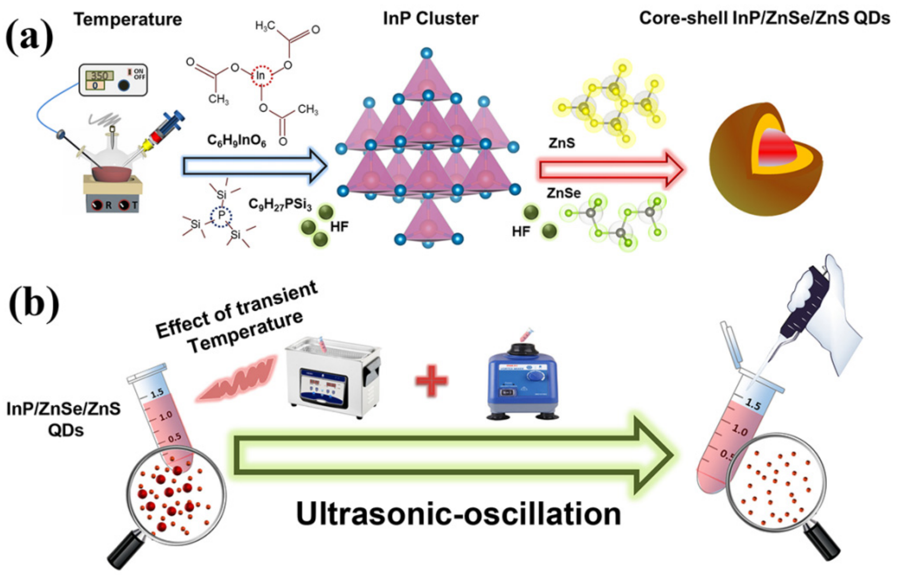

Fig. S1: InP/ZnSe/ZnS QDs. (a) Schematic illustration of the synthesis mechanism. (b) Schematic illustration of ultrasonic oscillation.

Characterization of InP/ZnSe/ZnS QDs

To explore the surface morphology of InP/ZnSe/ZnS QDs, TEM-based testing and analysis were performed. The black dots in Figure S2a are InP/ZnSe/ZnS QDs. The average lateral dimensions of InP/ZnSe/ZnS QDs (8.63 nm) were fitted. The FWHM was 0.32 nm (Figure S2b), and the QDs showed uniform monodispersity. Figure S2c shows a high-resolution TEM image of InP/ZnSe/ZnS QDs, where the lattice fringes are clearly observed. The lattice fringe spacing was 2.07, 2.38, and 2.26 Å for InP, ZnSe, and ZnS, respectively. The inset shows the diffraction spot of InP/ZnSe/ZnS QDs obtained from the fast Fourier transform analysis, revealing a cubic/hexagonal crystal structure. Figures S2d and S2e show the line profiles of the lattice fringes corresponding to the lattice spacings of InP, ZnSe, and ZnS in Figure 1c. Atomic force microscopy

* **Corresponding authors: Bin Yu,** Shenzhen Key Laboratory of Photonics and Biophotonics, Key Laboratory of Optoelectronic Devices and Systems of Ministry of Education and Guangdong Province, College of Physics and Optoelectronic Engineering, Shenzhen University, Shenzhen 518060, China, E-mail: yubin@szu.edu.cn. **Huiqun Cao,** College of Chemistry and Environmental Engineering, Shenzhen University, Shenzhen 518060, China, E-mail: chq0524@163.com. **Danying Lin,** Shenzhen Key Laboratory of Photonics and Biophotonics, Key Laboratory of Optoelectronic Devices and Systems of Ministry of Education and Guangdong Province, College of Physics and Optoelectronic Engineering, Shenzhen University, Shenzhen 518060, China, E-mail: dylin@szu.edu.cn.
Liangliang Zhou, Lilin Huang, Yingying Jing, Meiqin Wang, and Junle Qu, Shenzhen Key Laboratory of Photonics and Biophotonics, Key Laboratory of Optoelectronic Devices and Systems of Ministry of Education and Guangdong Province, College of Physics and Optoelectronic Engineering, Shenzhen University, Shenzhen 518060, China.

was used to determine the particle height. Figure S2f shows the three-dimensional height distribution of the InP/ZnSe/ZnS QDs. The average height distribution was 8.92 nm. Compared with the TEM characterization results, the InP/ZnSe/ZnS QDs showed a nearly spherical structure and morphology, likely improving the fluorescence quantum yield of the QDs. We tested the solution (0.005 mg/mL) under natural and ultraviolet light, and the color of the solution showed obvious changes (white and red), as shown in Figure S2g. Figure S2h shows the Raman spectrum of InP/ZnSe/ZnS QDs. The peaks at 238, 321, 353, 439, 502, 590, and 684 cm^{-1} correspond to ZnS/ZnSe, InP, InP/ZnS, double ZnS, silicon substrate, vibration of double InP/ZnS, and double InP molecular vibration, respectively, being consistent with previous reports [1, 2].

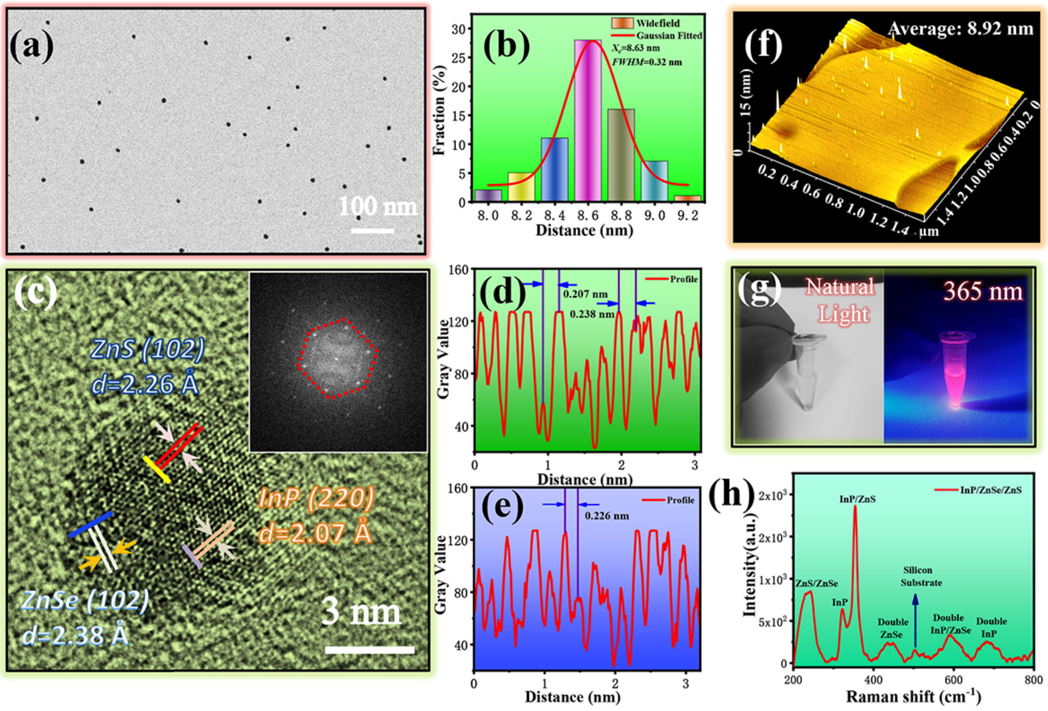

Fig. S2: Characterization of InP/ZnSe/ZnS QDs. (a) TEM image. (b) QD particle size distribution. (c) High-resolution TEM image of green and fast Fourier transform pattern (inset). (d, e) Line profile of diffraction fringes. (f) Morphology obtained from atomic force microscopy and average height. (g) QD photographs under natural light and 365 nm ultraviolet light. (h) Raman vibration spectrum.

To analyze the fluorescence properties, Figure S3a shows the absorption spectra of the InP cluster. We observed the InP cluster, and the 10, 15, 20, and 35 min valley–peak ratios of absorption were 531 nm/0.41, 538 nm/0.44, 547 nm/0.49, and 573 nm/0.52, respectively, indicating that the wavelength increased over time. In addition, the emission wavelengths of the InP/ZnSe/ZnS QDs were 620 and 624 nm (ultrasound and oscillation), as shown in Figure S3b. Moreover, the InP/ZnSe/ZnS QDs FWHM values were 620 nm (37.3 nm) and 624 nm (29.7 nm). The HF etching passivation and shell encapsulation of quantum dots can reduce oxygen interference, and thus improve the narrowband of quantum dots. The FWHM values were substantially reduced in the PL spectrum owing to ultrasound and oscillation for approximately 3 h, and high fluorescence quantum efficiency was maintained (620 nm–87.5% and 624 nm–100%). The temperature change of acoustic cavitation may cause certain defects, such as dangling bonds and slips on the surface of the nanoparticles via ultrasound and oscillation, which may enhance the fluorescence fluctuations. To verify the effect of temperature, we used Raman spectroscopy to analyze the PL spectra for different temperatures, as shown in Figure S3c. The wavelength increased (607–633 nm) with increasing temperature (−193–53 °C) in Figure S3d, and a decreasing trend in fluorescence intensity was observed.

First-principles simulations of the band gap of InP/ZnSe/ZnS QDs were performed using density functional theory implemented in the Vienna ab initio simulation package [3]. The electronic exchange-correlation effects were treated using Heyd–Scuseria–Ernzerhof (HSE) hybrid functionals [4, 5].

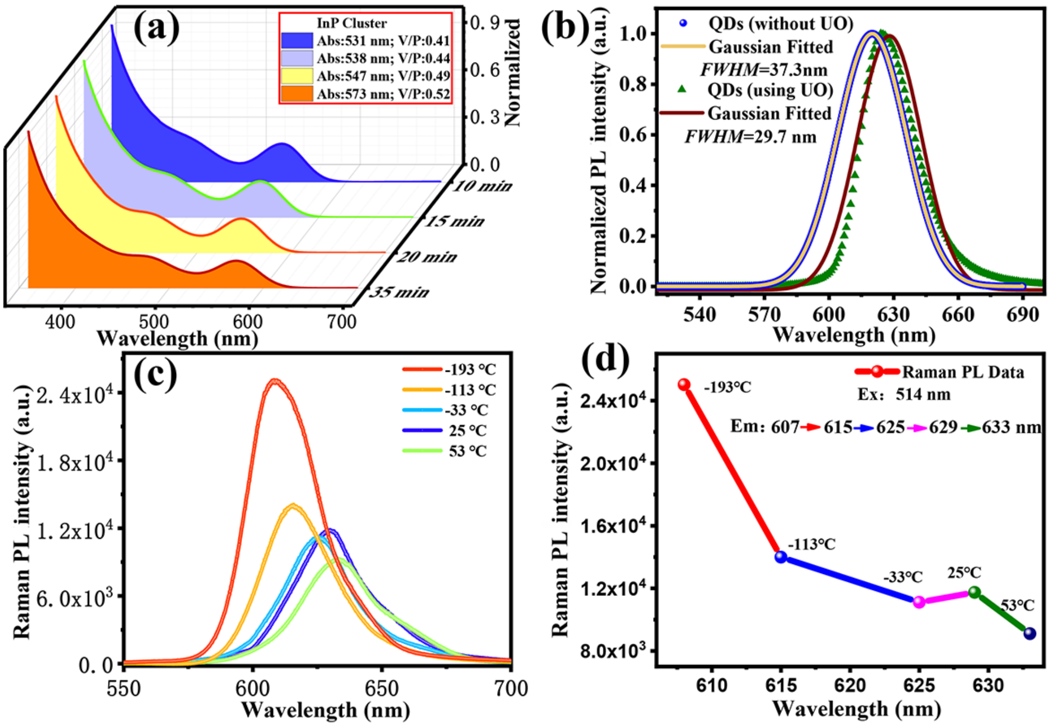

Fig. S3: Response of InP/ZnSe/ZnS QDs. (a) Absorption spectra. (b) PL spectra (QDs at 620 nm and QDs at 624 nm—ultrasound and oscillation). (c) Raman PL spectra at different temperatures and (d) spectrum peaks.

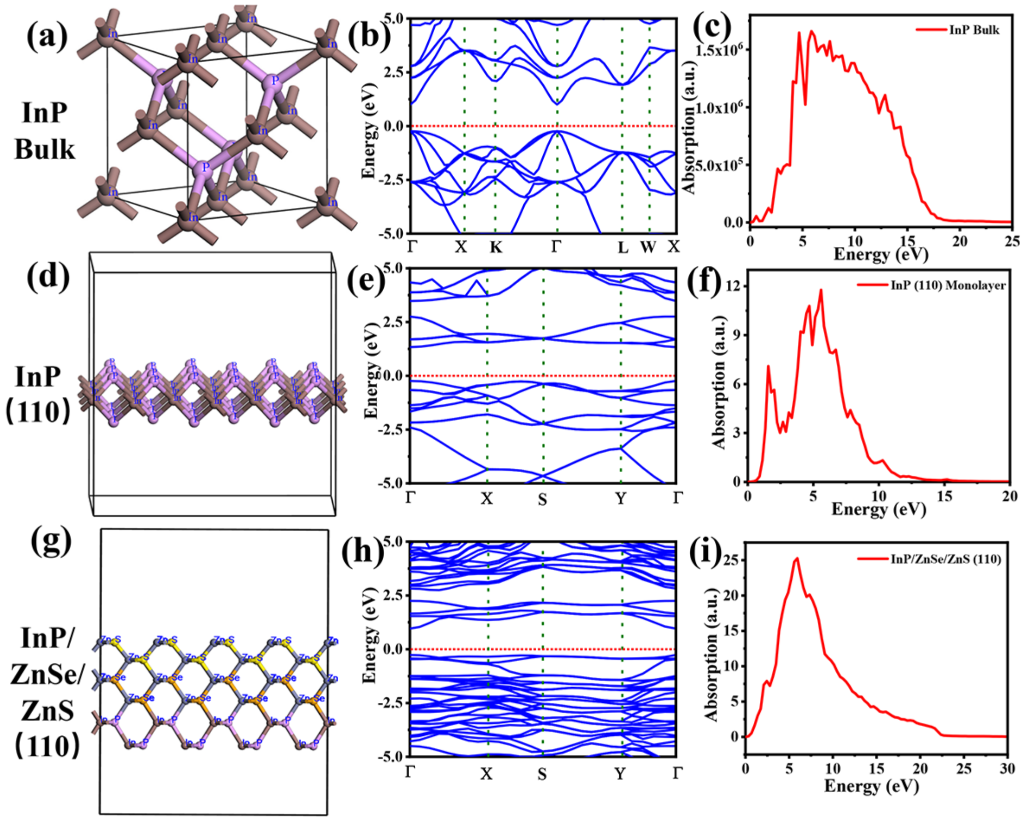

Fig. S4: First-principles calculation results. (a) Structure of bulk InP. (b) Band structure calculations of bulk InP by HSE06 functional. (c) Optical absorption spectra of bulk InP. (d) Structure of monolayer InP (110). (e) Band structure calculations of monolayer InP (110) by HSE06 functional. (f) Optical absorption spectra of monolayer InP (110). (g) Structure of InP/ZnSe/ZnS (110). (h) Band structure calculations of InP/ZnSe/ZnS (110) by HSE06 functional. (i) Optical absorption spectra of InP/ZnSe/ZnS (110).

In addition, 25% Hartree–Fock and 75% PBE–GGA were chosen for the short-range exchange part in the HSE06 hybrid functionals. The energy cutoff of the plane-wave basis was set to 520 eV [6]. The convergence criterion of energy was set to 1×10^{-5} eV during electronic structure calculations. For the structures of bulk InP in Figure S4a, a *k*-point grid mesh of 7 × 7 × 7 was used to sample the Brillouin zone. For the structures of the InP (110) and InP/ZnSe/ZnS (110) surfaces in Figures S4d and S4g, a vacuum region thickness of 15 Å was chosen to eliminate the interaction effects of the neighboring slab along the *Z* axis. A *k*-point grid mesh of 7 × 7 × 1 was used to sample the Brillouin zone of InP (110) and InP/ZnSe/ZnS (110) [7]. The band gaps of bulk InP (Figure S4b), InP (110) (Figure S4e), and InP/ZnSe/ZnS (110) (Figure S4(h)) were 1.24, 1.57, and 1.23 eV, respectively. These are direct band gaps at the Γ point. These results are in good agreement with those of previous studies [8, 9]. The bulk material exhibited an energy increase (wavelength blue-shift) to the monolayer and energy reduction (wavelength red-shift) from InP (110) to InP/ZnSe (110). Therefore, the InP wavelength could be stably tuned by increasing the imaging time. In addition, we calculated the optical absorption spectrum by simulating the real and imaginary parts, as shown in Figures S4c, S4f, and S4i, resulting in an absorption peak for 0–2 eV. The calculated optical band gap was approximately 2 eV considering the experimental spectra of InP/ZnSe/ZnS QDs (Figure S3b). This may be due to the change in the band gap from the Γ to the Y point by different atomic orbitals (Figure S4h) [10]. We found that the in-plane/out-of-plane vibration of the material may be intensified to generate dangling/dangling bonds through ultrasound and oscillation (Figure S2h) [11]. This may be an important factor affecting fluorescence blinking.

To further explore the effect of atomic orbitals on the band gap and fluorescence blinking, we analyzed the normalized absorption spectrum of InP/ZnSe/ZnS QDs, identifying peaks in 420–560 nm (optical band gap, 2–3 eV), as shown in Figure S5a. The partial density of states was calculated for each element of InP/ZnSe/ZnS (110), In, P, Zn, Se, and S (Figures S5b–S5f). Each element was occupied by s, p, and d orbitals. We focused on the influence of energy in 2–3 eV. We mainly observed Zn, Se, and S, and their orbitals were mainly s and p (Figures S5d–S5f). In addition, the orbital influence was more severe when the energy blue-shifts displaced to 2 eV. Therefore, it was possible to construct surface defects in InP/ZnSe/ZnS QDs through ultrasound and oscillation to obtain photoblinking properties.

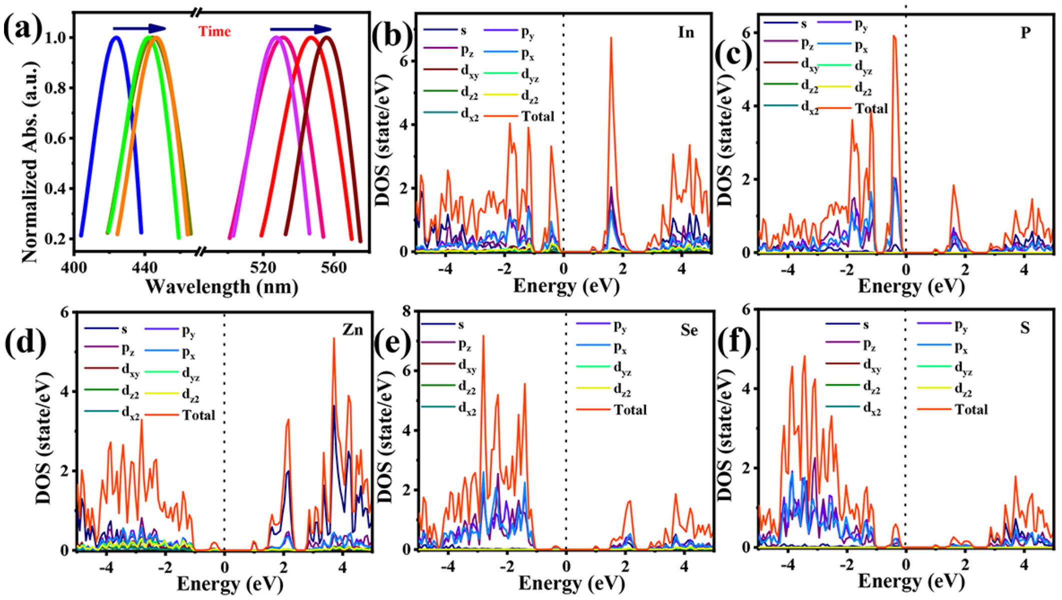

Fig. S5: (a) Normalized absorption spectrum of InP/ZnSe/ZnS QDs representing optical band gap of approximately 2–3 eV. (b)–(f) Partial density of states (DOS) for InP/ZnSe/ZnS (110), In, P, Zn, Se, and S, respectively.

Fluorescence Photoblinking at Other Exposure Times

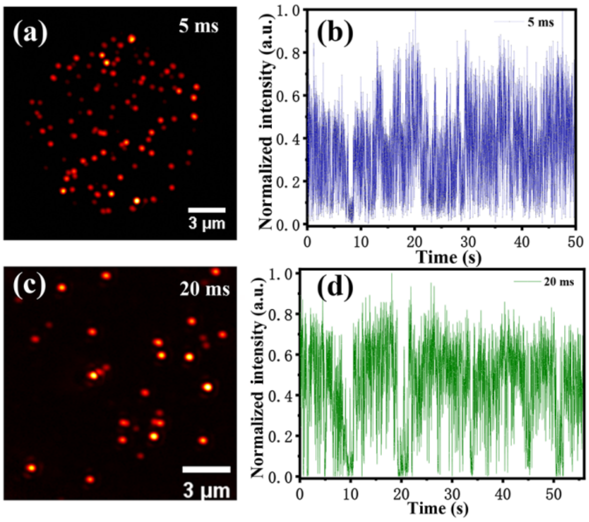

Fig. S6: (a) Single-particle widefield overlay image (10,000 frames). (b) PL fluctuation trace (5 ms). (c) Single-particle widefield overlay image (3000 frames). (d) PL fluctuation trace (20 ms).

Microtubule SOFI-MSIM Images at Different Frame Rates per Array

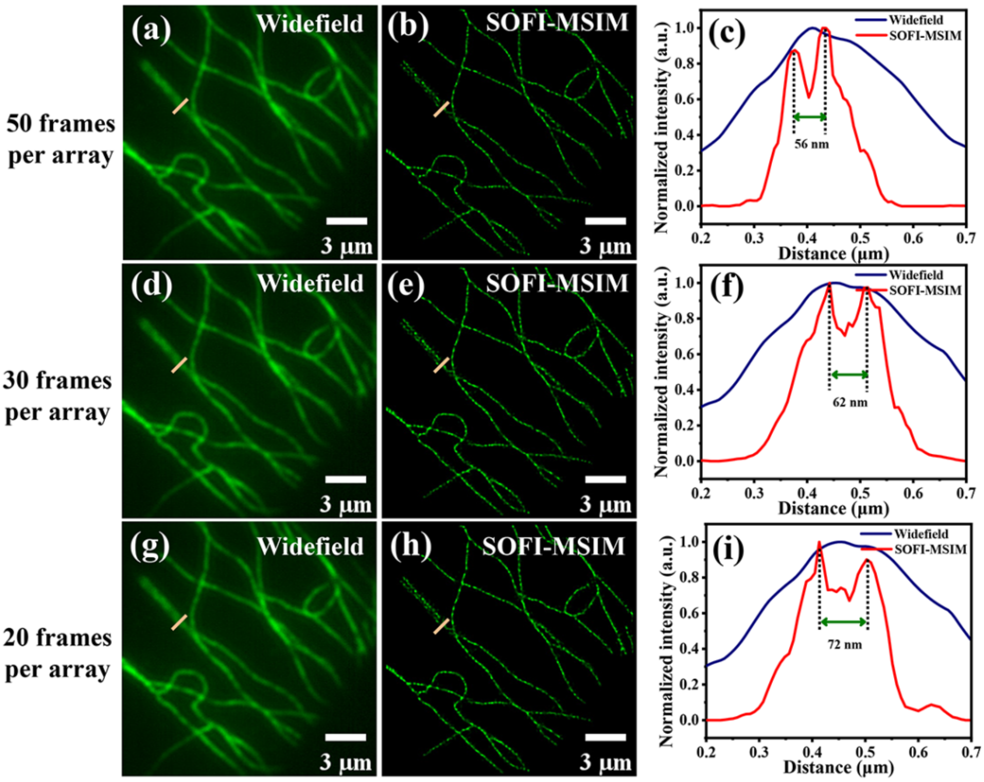

Fig. S7: Microtubules acquired using SOFI-MSIM (120 arrays). (a) Widefield and (b) SOFI-MSIM images (for 50 frames per array overlay) and (c) profile diagram. (d) Widefield and (e) SOFI-MSIM images (for 30 frames per array overlay) and (f) profile diagram. (g) Widefield and (h) SOFI-MSIM images (for 20 frames per array overlay) and (i) profile diagram.

Mitochondrial SOFI-MSIM Images at Different Frame Rates per Array

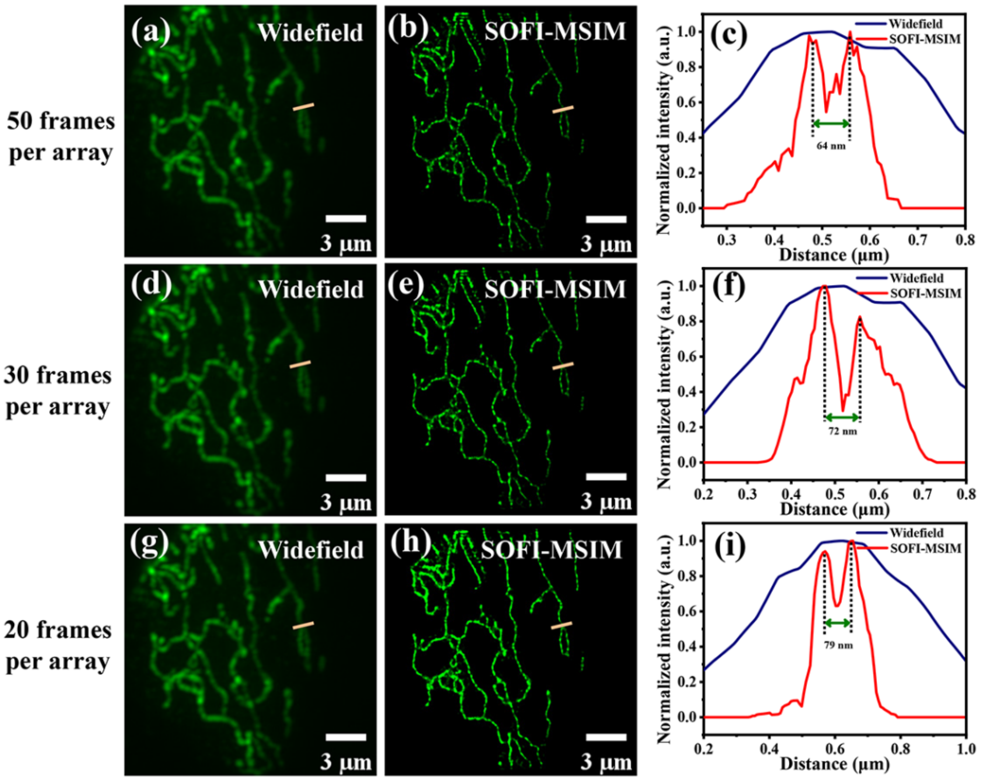

Fig. S8: Mitochondria acquired using SOFI-MSIM imaging (120 arrays). (a) Widefield and (b) SOFI-MSIM images (for 50 frames per array overlay) and (c) profile diagram. (d) Widefield and (e) SOFI-MSIM images (for 30 frames per array overlay) and (f) profile diagram. (g) Widefield and (h) SOFI-MSIM images (for 20 frames per array overlay) and (i) profile diagram.

SOFI and MSIM Imaging of InP/ZnSe/ZnS QD Fluorescent Probes

We labeled fixed BS-C-1 cell microtubules using InP/ZnSe/ZnS QD-SA. First, we performed SOFI imaging (1000 frames over approximately 5 s) and MSIM imaging (224 frames over approximately 1.12 s) on the MSIM system using a 488 nm laser excitation wavelength. Figures S9a and S9b show super-resolution microtubule images acquired using widefield imaging and SOFI. We analyzed the FWHM by Gaussian fitting (Figures S9e and S9f), obtaining 301 and 166 nm, respectively. Figures S9c and S9d show super-resolution microtubule images acquired using widefield imaging and MSIM. We analyzed the FWHM by Gaussian fitting (Figures S9g and S9h), obtaining 326 and 117 nm, respectively. Hence, the imaging resolution of SOFI or MSIM was improved by only two times. The proposed SOFI-MSIM maximized the image resolution under a large Stokes shift of InP/ZnSe/ZnS QD fluorescent probes.

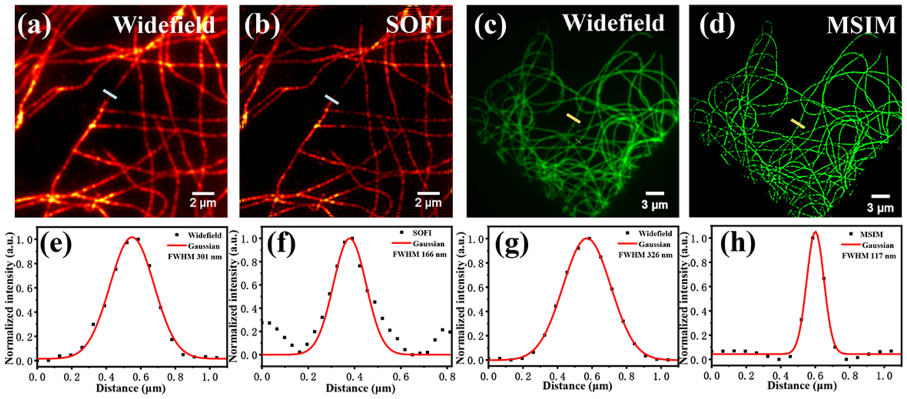

Fig. S9: Separate SOFI and MSIM. (a) SOFI widefield overlay image (1000 frames). (b) SOFI super-resolution image using Fourier ptychography algorithm. (c) MSIM widefield overlay image (224 frames). (d) MSIM super-resolution image using pixel relocation algorithm. FWHM values for (e) widefield imaging and (f) SOFI (highlighted in gray in panels a and b). FWHM values for (g) widefield imaging and (h) MSIM (highlighted in yellow in panels c and d).

References

[1] A. Brodu, M. V. Ballottin, J. Buhot, et al., “Exciton-phonon coupling in InP quantum dots with ZnS and (Zn, Cd) Se shells,” *Phys. Rev. B,* vol. 101, no. 12, p. 125413, 2020. https://doi.org/10.1103/PhysRevB.101.125413

[2] Y. H. Suh, S. Lee, S. M. Jung, et al., “Engineering Core Size of InP Quantum Dot with Incipient ZnS for Blue Emission,” *Adv. Opt. Mater.,* vol. 10, no. 7, p. 2102372, 2022. https://doi.org/10.1002/adom.202102372

[3] G. Kresse, J. Furthmüller, “Efficient iterative schemes for ab initio total-energy calculations using a plane-wave basis set,” *Phys. Rev. B*, vol. 54, no. 16, p. 11169, 1996. https://doi.org/10.1103/PhysRevB.54.11169

[4] J. Heyd, G. E. Scuseria, M. Ernzerhof, “Hybrid functionals based on a screened Coulomb potential,” *J. Chem. Phys.*, vol. 118, no. 18, pp. 8207–8215, 2003. https://doi.org/10.1063/1.1564060

[5] J. Heyd, G. E. Scuseria, “Efficient hybrid density functional calculations in solids: Assessment of the Heyd–Scuseria–Ernzerhof screened Coulomb hybrid functional,” *J. Chem. Phys.*, vol. 121, no. 3, pp. 1187–1192, 2004. https://doi.org/10.1063/1.1760074

[6] H. Yoo, K. S. Lee, S. Nahm, et al., “Predicting ligand-dependent nanocrystal shapes of InP quantum dots and their electronic structures,” *Appl. Surf. Sci.,* vol. 578, p. 151972, 2022. https://doi.org/10.1016/j.apsusc.2021.151972

[7] A. Jalil, Z. Zhuo, Z. Sun, et al., “A phosphorene-like InP3 monolayer: Structure, stability, and catalytic properties toward the hydrogen evolution reaction,” *J. Mater. Chem. A,* vol. 8, no. 3, pp. 1307–1314, 2020. https://doi.org/10.1039/C9TA08612A

[8] G. Garcia, P. Sanchez-Palencia, P. Palacios, et al., “Transition metal-hyperdoped InP semiconductors as efficient solar absorber materials,” *Nanomaterials,* vol. 10, no. 2, p. 283, 2020. https://doi.org/10.3390/nano10020283

[9] G. Xu, S. Zeng, B. Zhang, et al., “New generation cadmium-free quantum dots for biophotonics and nanomedicine,” *Chem. Rev.,* vol. 116, no. 19, pp. 12234–12327, 2016. https://doi.org/10.1021/acs.chemrev.6b00290

[10] S. Kang, Y. Kim, E. Jang, et al., “Fundamental limit of the emission linewidths of quantum dots: an ab initio study of CdSe nanocrystals,” *ACS Appl. Mater. Interfaces,* vol. 12, no. 19, pp. 22012–22018, 2020. https://doi.org/10.1021/acsami.0c02904

[11] O. Pagès, A. V. Postnikov, M. Kassem, et al., “Unification of the phonon mode behavior in semiconductor alloys: Theory and ab initio calculations,” *Phys. Rev. B*, vol. 77, no. 12, p. 125208, 2008. https://doi.org/10.1103/PhysRevB.77.125208
